# Corrigendum: Crystallizing highly-likely subspaces that contain an unknown quantum state of light

**DOI:** 10.1038/srep43982

**Published:** 2017-03-23

**Authors:** Yong Siah Teo, Dmitri Mogilevtsev, Alexander Mikhalychev, Jaroslav Řeháček, Zdeněk Hradil

Scientific Reports 6: Article number: 3812310.1038/srep38123; published online: 12 01 2016; updated: 03 23 2017

In this Article, an additional affiliation for Dmitri Mogilevtsev has been omitted. The correct affiliations for this author are listed below:

Institute of Physics, Belarus National Academy of Sciences, F. Skarina Ave. 68, 220072 Minsk, Belarus.

Centro de Ciências Naturais e Humanas, Universidade Federal do ABC, Santo André, SP, 09210-170 Brazil.

